# From Uncertainty to Competence: A Longitudinal Study of Confidence Development in Occupational Therapy Education

**DOI:** 10.1155/oti/1797008

**Published:** 2025-12-23

**Authors:** Natalie A. Perkins, Rajvinder Bains

**Affiliations:** ^1^ Department of Occupational Therapy, University of the Pacific, Sacramento, California, USA, pacific.edu

## Abstract

Confidence plays a critical role in students′ academic performance, particularly in complex foundational content such as neuroscience. While confidence is often discussed interchangeably with self‐efficacy, the two constructs are distinct yet complementary. Few studies have examined how confidence changes over time in occupational therapy neuroscience courses or what factors contribute to its development. This longitudinal mixed‐methods study examined confidence changes throughout a 15‐week neuroscience course in an Occupational Therapy Doctorate program. The study integrated quantitative measurements with qualitative reflections to understand both the magnitude and mechanisms of confidence development. Data were collected from 73 students across two independent cohorts in consecutive years (2023: *n* = 37; 2024: *n* = 36). Students completed weekly surveys with four Likert scale questions assessing neuroscience‐related confidence and three open‐ended questions at baseline, mid‐course, and end‐of‐course exploring factors influencing confidence. Analysis included nonparametric statistical tests for quantitative data and thematic analysis with sentiment analysis for qualitative responses. Both cohorts demonstrated statistically significant confidence increases from baseline to end‐of‐course (*p* < 0.001). Qualitative analysis revealed four themes replicating across both years: prior academic experiences (mixed effect), instructional support and learning environment (facilitator), interest in neurological practice (facilitator), and content complexity with anticipatory anxiety (barrier). Students attributed confidence gains to hands‐on learning activities, peer collaboration, and structured feedback. Sentiment patterns showed predominantly positive baseline attitudes despite concerns, shifting to more neutral mid‐course assessments reflecting realistic recalibration, then highly positive end‐of‐course reflections on self‐assessment value. Integration of quantitative and qualitative findings revealed that confidence development follows predictable patterns influenced by cognitive and emotional factors. Replication across two independent cohorts strengthens validity. Implications for occupational therapy education include intentional scaffolding, active learning opportunities, peer collaboration, and attention to Generation Z learners′ needs for external validation and structured support. These findings inform curriculum design strategies to enhance confidence, self‐efficacy, and clinical reasoning preparation.

## 1. Introduction

The relationship between student confidence and academic performance has long been a topic of interest in educational research, as confidence significantly influences motivation, engagement, and learning outcomes [1]. Confidence, defined as the belief in oneself and one′s abilities, can act as both a predictor of academic achievement and a mediating factor in skill acquisition [1]. However, while confidence may be correlated with self‐efficacy, defined as an individual′s judgment of their ability to perform specific tasks, the two concepts are distinct [1, 2].

Self‐efficacy focuses on perceptions related to specific tasks, whereas confidence encompasses a broader emotional state. Nevertheless, both constructs play crucial roles in shaping students′ learning experiences and outcomes.

Confidence is influenced not only by individual capabilities but also by contextual and emotional factors, including test anxiety, which is characterized by worry, interference, and emotionality and can significantly impact students′ confidence and academic performance [3]. Students with heightened anxiety may struggle with intrusive thoughts, diminished self‐esteem, and physiological responses that interfere with their ability to perform tasks. Academic self‐concept and self‐efficacy, both components of self‐directed competence beliefs, are closely linked to confidence and play critical roles in mitigating the negative effects of anxiety on academic performance [3].

This study was conducted to examine changes in students′ perceived confidence levels at different points during a trimester and the accompanying learning activities based on the following research questions: (1) What are the key moments of significant changes in confidence levels over the course of the term? (2) What are students′ baseline perceptions based on?

We hypothesized that students′ confidence in neuroscience courses would increase over the course of the term, with significant improvements following key instructional interventions, such as active learning activities and assessments.

## 2. Materials and Methods

### 2.1. Literature Review

Today′s graduate students are predominantly made up of Gen Z students, defined as individuals who were born between 1995 and 2010 [4] and grew up with instant gratification and a higher need for external validation, including praise and higher grades [5].

This generation′s self‐esteem is positively correlated with self‐efficacy [6]. This means that individuals with high self‐esteem are more likely to believe in their ability to successfully perform tasks and achieve their goals, which, in turn, enhances motivation, persistence, and overall academic or professional performance.

This relationship between self‐esteem and self‐efficacy is particularly relevant in academic settings, as Gen Z students seek frequent feedback and external reinforcement to validate their abilities [5]. Research suggests that students who receive consistent and constructive feedback develop a stronger sense of competence, further enhancing their self‐efficacy and academic resilience [7]. However, overreliance on external validation may also pose challenges, leading to decreased intrinsic motivation and increased anxiety when faced with critical assessments or independent problem‐solving tasks [8, 9]. Therefore, fostering a balanced approach that encourages both self‐regulation and external support is essential for optimizing learning outcomes and confidence development among graduate students.

### 2.2. Theoretical Frameworks

Two theoretical frameworks were used to develop this research project to better understand the foundational components of the identified problem or knowledge gap. This gap lies in the decreased understanding of how students′ perceived confidence in neuroscience‐related competencies evolves and its relationship with academic performance in occupational therapy education. While confidence and self‐efficacy affect learning outcomes, limited research has been conducted to examine explicitly how students develop confidence in locating neurological structures, learning neuroscience content, applying neurological concepts to occupational therapy practices, and applying knowledge in case studies. The social cognitive theory (SCT) [10] and self‐validation theory (SVT) [11] guided the development of this study, emphasizing the relationship between confidence and student learning.

These confidence dimensions were investigated over a 15‐week term across 2 years in two cohorts to bridge the gap between theoretical knowledge and practical applications in neuroscience education for occupational therapy students. The findings will inform curriculum design, instructional strategies, and student support mechanisms to enhance confidence and competency development in this challenging subject area.

SCT refers to the mental process of perceiving and responding to social information. Bandura [10] developed the SCT framework to understand the interplay between environmental influences, personal factors, and behavior. This theory emphasizes the reciprocal relationship between personal factors, the environment, and behaviors. This theory guided the present study in understanding how students′ confidence is shaped by their academic experiences, prior successes, and external factors, such as instructors and prior student experiences.

SVT refers to mental beliefs that guide overall daily attitudes and decisions. Briñol and Petty [11] explained how perceived thought validity influences judgments and actions. In the present study, this theory explains how students′ confidence levels affect their reliance on their thoughts when learning new concepts. The theory highlights that confidence is influenced by academic competence and external factors such as mood, stress, and prior experiences.

In summary, the SCT and SVT provide a lens for examining the internal and external factors that impact students′ perceived confidence levels. Understanding the impact of students′ internal thoughts and beliefs can help educators develop targeted strategies to foster confidence and enhance self‐efficacy.

### 2.3. Research Design

This study utilized a longitudinal mixed‐methods convergent design to examine changes in perceived confidence levels among second‐year occupational therapy doctoral students throughout a 15‐week neuroscience course. The mixed‐methods approach integrated quantitative confidence measurements (Likert scale surveys) with qualitative reflections (open‐ended responses) to provide a comprehensive understanding of both the magnitude of confidence changes and the underlying factors contributing to these changes. This design was conducted across two independent cohorts in consecutive academic years (2023 and 2024) to assess the replicability of findings and strengthen the validity of results.

### 2.4. Setting and Context

The study was conducted at a single university in California within an Occupational Therapy Doctorate program. The neuroscience course was a 15‐week, trimester‐based course covering neuroanatomy, neurophysiology, and neurorehabilitation as applied to occupational therapy practice.

The course employed multiple instructional strategies throughout the term:

• Traditional lectures with PowerPoint presentations (approximately 40% of course time)
• Hands‐on laboratory activities for neuroanatomical structure identification (approximately 25% of course time)
• Case‐based learning applications integrating neurological concepts with occupational therapy practice (approximately 20% of course time)
• Collaborative group assignments and peer discussion activities (approximately 10% of course time)
• Formative assessments including weekly quizzes and knowledge checks (approximately 5% of course time)
• Soft chalk interactive learning modules for self‐paced review
• Clinical application exercises linking neuroscience content to occupational therapy interventions

Both cohorts were taught by the same instructors (the study authors) using consistent curriculum content, instructional strategies, and timing of pedagogical interventions across both academic years to ensure comparability of findings.

### 2.5. Participants and Sampling

Ethical approval was granted by the university′s institutional review board prior to data collection. Convenience sampling was utilized to recruit participants from two consecutive cohorts of second‐year occupational therapy graduate students enrolled in the neuroscience course. A total of 73 students participated in the study: 37 students from the 2023 cohort (Class 1) and 36 students from the 2024 cohort (Class 2). All participants provided informed consent after being informed that participation was voluntary, anonymous, and would have no impact on their course grades or academic standing.

### 2.6. Researcher Positionality and Bias Mitigation

Because the course instructors (the study authors) were also the researchers, several measures were implemented to minimize potential bias and coercion:

1. Participation was explicitly voluntary with no academic consequences for nonparticipation or withdrawal; students could opt out of any survey at any time.
2. All surveys were administered anonymously with no identifying information collected that could link responses to individual students.
3. Instructors had no knowledge of which students completed surveys or their individual responses; the anonymous survey system prevented instructor access to participant‐level data during the course.
4. Data analysis was conducted after final course grades were submitted for each cohort.
5. A research assistant who was not involved in course instruction assisted with data collection and preliminary analysis.
6. Students were reminded at multiple timepoints that they could withdraw from the study at any time without penalty.
7. Survey responses were not reviewed by instructors until after the completion of each course to prevent any influence on grading or instructional decisions.

These measures were designed to create an ethical research environment that protected student autonomy while allowing for meaningful data collection on confidence development. The instructor blinding (measure #3) was particularly important in preventing any potential influence (conscious or unconscious) on student interactions, grading, or instructional decisions based on survey participation or responses.

### 2.7. Data Collection

#### 2.7.1. Survey Instrument Development

The survey instrument was developed based on SCT and SVT frameworks, focusing on domain‐specific confidence measures relevant to neuroscience competencies in occupational therapy education. The quantitative questions were designed to assess confidence in specific, observable competencies identified as critical for neuroscience learning in occupational therapy programs. The qualitative questions were designed to capture students′ reflections on factors influencing their confidence and their metacognitive awareness of confidence development.

#### 2.7.2. Survey Validation

Prior to implementation, the survey questions were reviewed by three content experts in occupational therapy education for face validity, clarity, and alignment with course learning objectives. Feedback from expert review was incorporated to refine question wording and ensure questions appropriately captured the constructs of interest. However, the survey instrument was not formally validated through comprehensive psychometric testing (including test–retest reliability, construct validity, or factor analysis). This limitation is acknowledged and discussed in the limitations section.

### 2.8. Survey Components

The mixed‐methods survey instrument consisted of both quantitative and qualitative components administered via an online platform. Each survey took approximately 5 min to complete.

#### 2.8.1. Quantitative Component

Students responded to four Likert scale questions assessing confidence in specific neuroscience‐related competencies:

1. “How confident are you in your ability to locate neurological structures?”
2. “How confident are you in your ability to learn the neuroscience course content?”
3. “How confident are you in your ability to apply neurological concepts to occupational therapy practice?”
4. “How confident are you in your ability to apply knowledge of neurological functions to case study questions?”

Responses were rated on a 5‐point Likert scale: 1 = *strongly disagree*, 2 = *disagree*, 3 = *neutral*, 4 = *agree*, 5 = *strongly agree*. Higher scores indicated greater perceived confidence. The 5‐point scale was selected intentionally to eliminate a neutral midpoint and encourage students to lean toward either agreement or disagreement regarding their confidence levels.

#### 2.8.2. Qualitative Component

Three open‐ended questions were administered at strategic timepoints to capture students′ evolving reflections on confidence factors:

1. Week 1 (baseline): “What factors impact/contribute to your perceived level of confidence?”
2. Week 8 (mid‐course): “What factors over the trimester contributed to your perceived level of confidence?”
3. Week 15 (end‐of‐course): “How did you use the surveys to self‐reflect on your progress in the course?”

These open‐ended questions were designed to elicit rich, contextualized responses about students′ confidence perceptions, the factors they identified as influential, and their metacognitive awareness of confidence development throughout the course.

#### 2.8.3. Survey Administration Timeline

Surveys containing the quantitative component were administered weekly throughout the 15‐week course for both cohorts. Surveys were distributed electronically at the beginning of each class session, and students completed them anonymously within the first 5 min of class time. The first survey was administered during Week 1, and subsequent surveys were administered weekly through Week 15, providing 15 timepoints of quantitative data per student. The three qualitative open‐ended questions were embedded within the surveys at Weeks 1, 8, and 15 as specified above.

Response rates varied across the 15 weeks due to voluntary participation and student absences. For the analyses presented in this study, baseline confidence (Week 1) was compared to end‐of‐course confidence (Week 15) among students who completed both timepoints.

### 2.9. Data Analysis

#### 2.9.1. Quantitative Data Analysis

Quantitative confidence data were analyzed using both descriptive and inferential statistics. Means, standard deviations, and ranges were calculated for each survey question at baseline and end‐of‐course for both cohorts.

To examine overall changes in confidence from baseline (Week 1) to end‐of‐course (Week 15), nonparametric statistical tests were employed due to the ordinal nature of Likert scale data and concerns about participant attrition affecting paired‐sample analyses.

The Mann–Whitney *U* test was performed to compare baseline and final confidence scores between the two cohorts to determine whether confidence patterns differed between years. Subsequently, the Wilcoxon signed‐rank test was conducted to compare baseline and final confidence levels within each cohort among participants who completed both surveys. A one‐tailed test was conducted at the *α* = 0.05 significance level to test the directional hypothesis that confidence levels would improve over time.

### 2.10. Qualitative Data Analysis

Qualitative responses to open‐ended questions were analyzed using a combination of thematic analysis and sentiment analysis.

#### 2.10.1. Thematic Analysis

An inductive thematic coding approach was employed to identify recurring themes in student responses. To enhance the credibility of the qualitative analysis, data triangulation was utilized by having two independent researchers separately review and interpret survey responses to identify key themes. The analysis followed established qualitative methodology:

1. Both researchers independently read all qualitative responses multiple times to gain familiarity with the data.
2. Each researcher independently generated initial codes capturing key concepts mentioned by students.
3. Researchers independently identified patterns and clustered codes into preliminary themes.
4. The two researchers met to compare their independent analyses, discussing areas of agreement and divergence.
5. Through iterative discussion, researchers reached mutual consensus on the final thematic structure
6. Final themes were clearly defined with supporting evidence from student responses.

This consensus‐based approach ensured that identified themes were not idiosyncratic to a single researcher′s interpretation but represented patterns evident to multiple analysts.

#### 2.10.2. Sentiment Analysis

Sentiment analysis was conducted using the VADER (Valence Aware Dictionary and sEntiment Reasoner) sentiment analysis tool. VADER generates compound scores ranging from −1 (most negative) to +1 (most positive), along with proportions of negative, neutral, and positive sentiment for each response. Responses were categorized as predominantly negative (compound score < −0.05), neutral (−0.05 to 0.05), or positive (> 0.05) based on their compound scores.

#### 2.10.3. Mixed‐Methods Integration

Quantitative and qualitative data were integrated at the interpretation stage using a convergent mixed‐methods design approach. Qualitative themes were mapped to quantitative confidence patterns to explain observed trajectories. Qualitative data illuminated “why” confidence changed (mechanisms and contributing factors), while quantitative data demonstrated “how much” confidence changed (magnitude and statistical significance).

## 3. Results and Discussion

At the beginning of the term, the students in both Class 1 and Class 2, which represent two distinct cohorts of 34 students in 2023 and 36 students in 2024, reported low levels of perceived confidence in their subject matter, with a mean confidence rating of 1.54. By the end of the term, the mean confidence rating increased to 3.57 for Cohort 1 (Class 1) and 3.45 for Cohort 2 (Class 2), suggesting a marked improvement in students′ perceptions of personal competence.

To examine changes in confidence level over time, the Mann–Whitney *U* test was performed to compare baseline and final confidence scores between the two classes. This nonparametric test was selected because of the ordinal nature of the data and participant attrition, which limited the use of paired‐sample tests for the full dataset.

The Wilcoxon signed‐rank test was subsequently conducted to compare baseline and final confidence levels within each class among the participants who completed both surveys. A one‐tailed test was conducted at the *α* = 0.05 level to test the hypothesis that confidence level would improve over time.

For Class 1, the Wilcoxon test yielded a *z* value of −3.92 and a *p* value of 0.0004, indicating a statistically significant increase in confidence level. The *W* value was 0, with a critical *W* value of 60 at *p* < 0.05.

For Class 2, the *z* value was −4.20, with a *p* value of 0.00001, also indicating significance. The *W* value was again zero, with a critical *W* value of 83 at *p* < 0.05.

These findings align with previous research that highlights the relationship between structured learning environments and increased self‐efficacy [11]. Instructional strategies such as scaffolded activities, frequent feedback, self‐reflection, and collaborative learning have been shown to enhance confidence and engagement in learners [12]. The improvement in confidence across both cohorts suggests that the course design effectively supported skill development and the belief in one′s capabilities.

These results confirmed a statistically significant increase in students′ perceived confidence levels from the beginning to the end of the term in both classes (Table 1). Additional visual representations of these findings are presented in Supporting Information 1: Figure S1 and Supporting Information 2: Figure S2.

**Table 1:** Inferential statistics summary.

**Test**	**Class**	**z** **value**	**p** **value**	**W** **value**	**Critical** **W**
Wilcoxon′s signed‐rank test	Class 1	−3.92	0.0004	0	60
Wilcoxon′s signed‐rank test	Class 2	−4.20	0.00001	0	83

Supporting Information 1: Figure S1 illustrates the average student self‐reported confidence level in four neuroscience‐related competencies: locating neurological structures, learning neuroscience course content, applying neurological concepts to occupational therapy practice, and applying knowledge of functions to case study questions. Data are shown for Class 1 and Class 2. Responses were rated on a 5‐point Likert scale, with higher values indicating greater confidence levels. Students reported the highest confidence in learning neuroscience course content (light teal bars, Supporting Information 1: Figure S1), while confidence in applying knowledge to case studies (orange bars) showed more fluctuation.

Students′ confidence in knowledge of neuroscience content (Supporting Information 2: Figure S2) followed a similar trajectory across both cohorts, beginning at lower levels in Week 1 (mean ~2.5) and increasing substantially by Week 15 (mean ~3.5), with temporary dips corresponding to mid‐semester assessment periods.

### 3.1. Qualitative Findings

To better understand the students′ perceptions related to confidence in their neurological occupational therapy content, qualitative feedback from surveys was analyzed. To enhance the credibility of the qualitative analysis, data triangulation was utilized by having two independent researchers separately review and interpret survey responses to identify key themes. When the researchers compared their findings, there was mutual consensus on the final thematic structure. Analysis of responses to “What factors impact/contribute to your perceived level of confidence?” revealed four primary themes that explain students′ initial confidence perceptions. These themes emerged consistently across both the Class 1 (*n* = 16‐30 responses per question) and Class 2 (*n* = 29‐44 responses per question), demonstrating replication of findings. Three of the themes were identified as facilitators of confidence development, and one emerged as a barrier.

#### 3.1.1. Theme 1: Prior Academic Experiences (Facilitator With Mixed Effects)

Students′ previous coursework in neuroscience and related sciences significantly influenced their baseline confidence, though this influence was bidirectional. Those with positive prior experiences in anatomy, physiology, or neuroscience reported feeling better prepared and demonstrated higher initial confidence levels. As one student explained:

I majored in psychology and have a really strong interest in neurological disorders and the role in OT for treatment and recovery. (2024)

Prior knowledge served as a scaffold for learning, allowing students to build upon existing foundations. However, students who had struggled in previous science courses or lacked neuroscience background expressed lower initial confidence:

I have very little background education/knowledge in neuroscience aside from anatomy which I found challenging. (2024)

This theme appeared in 45% of 2024 baseline responses and was consistently present in 2023 responses, explaining the variability observed in baseline confidence scores (range: M = 1.54 to complete confidence). The bidirectional nature of prior experience highlights the importance of assessing students′ academic histories when designing supportive interventions.

#### 3.1.2. Theme 2: Instructional Support and Learning Environment (Facilitator)

Students consistently identified teaching methods, instructor support, and learning structure as key facilitators of confidence development. Collaborative in‐class discussions, hands‐on learning activities, and case‐based learning emerged as particularly valuable strategies. Students appreciated structured guidance and clear instructional frameworks:

Being that we have lectures and PowerPoints, I feel that this is how I learn best and I am glad that this course has that available. (2023)

I enjoyed all the hands‐on activities and SoftChalk modules provided to us. (2023)

Mid‐course reflections revealed that active learning experiences significantly contributed to growing confidence. Students specifically credited hands‐on laboratory activities, peer collaboration opportunities, and targeted formative assessments as building their competence. One student noted:

Peer support, targeted concepts on final that overlapped with what I studied, clear lectures. (2023)

The emphasis on structured support and explicit instructional frameworks aligns with Generation Z characteristics, as these learners tend to seek external validation and clear guidance. The consistency of this theme across both cohorts (appearing in 38% of baseline responses and increasing in mid‐course reflections) validates the importance of intentional instructional design in fostering confidence.

#### 3.1.3. Theme 3: Interest in Neurological Occupational Therapy Practice (Facilitator)

Despite concerns about course difficulty, many students expressed genuine interest in neuroscience applications to occupational therapy practice, which served as a powerful motivator and confidence‐building factor. Students who identified professional passion for neurological practice demonstrated higher engagement and confidence:

I have always had a strong interest in mental health and neurological disorders, especially in the role of OT in rehabilitation. This is an area that I can see myself going into in the future. (2023)

This intrinsic motivation and recognition of professional relevance helped students persevere through challenging content. Exposure to real‐life cases sparked curiosity and professional motivation, helping to bridge the gap between theoretical knowledge and clinical application. The clinical relevance of neuroscience content served as both a confidence facilitator and a sustaining factor throughout the course, appearing in 18% of responses across both cohorts.

#### 3.1.4. Theme 4: Content Complexity and Anticipatory Anxiety (Barrier)

The most frequently cited barrier to confidence was the perceived complexity and volume of neuroscience content, mentioned by 40% of 2024 respondents at baseline. Students expressed concerns about the sheer amount of material, the abstract nature of neurological concepts, and the challenge of integrating dense information:

The complexity and vast information there is around neuroscience makes this subject intimidating. (2024)

Anticipatory anxiety emerged as a significant subtheme, with students reporting anxiety even before encountering the course material. This anxiety stemmed from multiple sources: course reputation based on previous cohorts′ experiences, concerns about memorization demands, and worry about the perceived gap between textbook knowledge and clinical application:

I think that past experiences have made me struggle with allowing myself to learn more complex concepts. My anxiety can get in my way and I can get overwhelmed with the amount of reading and material to review. (2024)

Test anxiety was explicitly mentioned by multiple students across both cohorts, highlighting the psychological and emotional dimensions of confidence development beyond purely cognitive factors. This finding aligns with SVT, as anxiety appeared to reduce students′ confidence in their thought validity when approaching new material.

Interestingly, mid‐course reflections showed a shift in sentiment, with students developing more realistic, balanced assessments of their capabilities after experiencing course demands. While initial responses were 57% positive (2023) and 45% positive (2024), mid‐course responses shifted to more neutral sentiment (56% neutral in 2023, 46% neutral in 2024), suggesting healthy recalibration rather than sustained anxiety.

### 3.2. Cross‐Cohort Replication and Sentiment Analysis

The replication of these four themes across Class 1 and Class 2 strengthens the validity and generalizability of findings. Sentiment analysis revealed consistent patterns across years (Table 2).

**Table 2:** Sentiment analysis.

	**Baseline sentiment Week 1**	**Mid-course sentiment Week 8**	**End of course sentiment Week 15**
Class 1 (2023)	57% positive 23% negative 20% neutral (*n* = 30)	39% positive 6% negative 56% neutral (*n* = 18)	59% positive 6% negative 35% neutral (*n* = 17)
Class 2 (2024)	45% positive 23% negative 32% neutral (*n* = 44)	40% positive 14% negative 46% neutral (*n* = 35)	76% positive 10% negative 14% neutral (*n* = 29)

The shift from predominantly positive baseline sentiment (despite expressed concerns) to more neutral mid‐course sentiment suggests students developed more realistic self‐assessments after experiencing course demands. The highly positive end‐of‐course sentiment regarding self‐reflection value indicates students appreciated the metacognitive opportunities provided by weekly surveys.

### 3.3. Integration With Quantitative Findings

Together, these four themes highlight the possible mechanisms underlying the quantitative confidence increases observed in both cohorts (Class 1: *z* = −3.92, *p* = 0.0004; Class 2: *z* = −4.20, *p* = 0.00001). While quantitative measures demonstrated significant overall growth, qualitative analysis revealed that confidence development involved not only skill acquisition but also emotional regulation, cognitive reframing, and the development of self‐directed learning capabilities.

Students attributed their confidence gains to specific, replicable instructional strategies including hands‐on learning activities, peer collaboration, and structured feedback. These teaching strategies align with SCT′s emphasis on mastery experiences and social modeling as sources of self‐efficacy. The qualitative data also revealed psychological factors (anxiety management, self‐doubt, and gradual belief development) that quantitative measures alone could not capture, demonstrating processes central to SVT.

The convergence of quantitative and qualitative findings provides comprehensive evidence that confidence development in neuroscience education follows predictable patterns that educators can systematically support through intentional curricular design, appropriate scaffolding of complex content, and attention to both cognitive and emotional aspects of learning.

## 4. Limitations

This study has several limitations that may affect interpretation and generalizability of these findings. First, the research was conducted at a single university in California, which limits the generalizability of the findings to other occupational therapy programs. The neuroscience courses have characteristics that may differ from courses at other occupational therapy programs. The use of a convenience sample further narrows the applicability of results to broader populations. Regional factors, program selectivity, cohort size, and institutional resources could all influence how students experience neuroscience education. Additionally, participant attrition over the course of the trimester may have introduced bias, particularly in the inferential statistical analysis. The replication across two consecutive years at the same institution strengthens confidence in the patterns observed but does not establish that these patterns would appear at other institutions.

All data came from self‐report which may mean that students responded in socially desirable ways even though the surveys were anonymous. Some students may have hurried through responses, while others may have taken more time. The accuracy of self‐reported confidence depends on the students′ self‐awareness and honesty, both of which vary. While anonymous surveys reduce some of these factors, they cannot eliminate them entirely.

Future research could consider implementing similar confidence surveys in courses that are required across all doctoral‐level occupational therapy programs, which could increase both participation and national representation.

### 4.1. Implications for Occupational Therapy

Despite these limitations, this study has important implications for occupational therapy education. The findings highlight the need for educators to design intentional instructional strategies that support the development of student confidence and self‐efficacy, especially for mastering complex neuroscience content. Given that Gen Z students, who represent a substantial portion of the current occupational therapy cohorts, tend to seek external validation and frequent feedback [4, 5], educators may benefit from incorporating structured feedback opportunities and scaffolded mastery experiences into the curriculum.

By fostering educational environments that emphasize internal validation while addressing challenges such as test anxiety, occupational therapy programs can enhance students′ abilities to identify neurological structures, understand complex neuroscience materials, and apply this knowledge to clinical reasoning and case‐based learning. This balanced approach can lead to improved academic performance and better preparation for the clinical decision‐making required in professional practice.

## 5. Conclusion

This longitudinal study tracked how occupational therapy students′ confidence evolved during a 15‐week neuroscience course, examining both the degree of change and the reasons behind it. Student confidence increased significantly in both cohorts studied, moving from a mean of 1.54 at the start to 3.57 and 3.45 by course end. The consistency of these results across two different groups of students, taught in consecutive years, suggests the patterns observed are not anomalous but reflect genuine developmental trajectories that may occur in similar educational contexts.

What students said about their experiences helps explain the numbers. Those who entered the course with stronger science backgrounds or previous neuroscience exposure tended to start with more confidence, while those with limited preparation or past difficulties in related subjects expressed more uncertainty. The volume and complexity of neurological content intimidated many students initially, and anxiety about the course (often based on what they had heard from previous cohorts) affected their early perceptions of their own capabilities. Yet, alongside these concerns, most students expressed real interest in neurological practice within occupational therapy, which appeared to sustain their engagement even when the material felt overwhelming.

As the course progressed, students identified specific experiences that built their confidence. Hands‐on laboratory activities, where they could physically identify and manipulate neurological structures, came up repeatedly in their reflections. Working with peers helped both the collaborative assignments and informal study groups. Students also valued the structure provided through lectures, targeted feedback on assessments, and opportunities to apply neuroscience concepts to occupational therapy cases. These strategies align with established learning theory, particularly Bandura′s work on how mastery experiences and social modeling contribute to self‐efficacy. But the students′ own words make clear that what mattered most was when abstract neurological information connected to something tangible, they could see, touch, or apply to patient care.

The shift in how students talked about their confidence over time was particularly interesting. Early in the course, despite their worries, many students expressed optimism about succeeding. By mid‐semester, their language became more measured with fewer extremes of anxiety or confidence, more neutral assessments of where they stood. This recalibration likely reflects a more accurate understanding of both the course demands and their own capabilities after several weeks of actual work. Students were neither overconfident nor paralyzed by doubt; they had simply adjusted their expectations to match reality.

The value students placed on regular self‐reflection deserves mention. Many described using the weekly confidence surveys to monitor their own progress, identify areas needing more attention, and adjust their study approaches. This metacognitive awareness, which is thinking about their own thinking, may have contributed to confidence development independent of any instructional strategy. When students step back to assess their learning regularly, they gain perspective on their growth that might otherwise go unnoticed.

For occupational therapy educators, these findings suggest several practical considerations. First, student anxiety about neuroscience content is real and widespread, not limited to a few struggling individuals. Acknowledging this openly and providing early success experiences may help prevent anxiety from becoming a self‐fulfilling prophecy. Second, while lectures and readings provide necessary information, confidence appears to build most reliably through active engagement in labs, case discussions, peer work, and other formats where students do something with the knowledge rather than simply receive it. Third, the scaffolding of material matters, particularly for students without strong science preparation. Starting with foundational concepts and building systematically toward complexity may reduce the sense of being overwhelmed that many students described.

The Generation Z students in these cohorts showed characteristics documented in other research on this population: They sought external validation through feedback, preferred structured learning environments over completely self‐directed approaches, and valued clear expectations. They were also remarkably self‐aware about their own anxiety and learning needs, often articulating quite specifically what helped or hindered their confidence. Instructors working with similar student populations might consider their need for regular checkpoints and explicit guidance not as a deficit but as a learning preference that can be accommodated while still maintaining academic rigor.

Some limitations warrant consideration. This study took place at one university with a specific curriculum and teaching approach, so results may not transfer directly to other settings. The survey questions, while reviewed by content experts, were not formally validated through psychometric testing. Students self‐reported their confidence, which may or may not correspond to actual competence since feeling confident and being competent are related but distinct. Participation was voluntary and anonymous, which protected students but meant we could not track individual trajectories or connect confidence changes to academic performance. Some students stopped completing surveys as the term progressed, and it is possible that those who persisted differed systematically from those who did not.

Despite these constraints, the study offers a window into how confidence develops when students encounter difficult material that is directly relevant to their future professional work. The replication of findings across two independent groups strengthens the case that these patterns have some generality. Future research might examine whether confidence at different points in the course predicts subsequent academic performance or clinical competence. It would also be valuable to know whether the confidence students build in neuroscience courses carries over into their clinical rotations and, ultimately, into practice.

Confidence alone does not make a competent occupational therapist, but students who believe they can learn complex material, apply it appropriately, and continue developing their knowledge throughout their careers are better positioned to do so. The neuroscience course represents just one part of professional preparation, but it is a part that many students find particularly challenging. Understanding how students navigate that challenge, including what helps, what hinders, and how their perceptions shift over time, can inform how occupational therapy programs structure these courses to support both learning and the development of professional self‐assurance.

## Conflicts of Interest

The authors declare no conflicts of interest.

## Funding

No funding was received for this manuscript.

## Supporting Information

Additional supporting information can be found online in the Supporting Information section.

## Supporting information


**Supporting Information 1** Figure S1: Student self‐reported confidence in neuroscience‐related competencies across Class 1 and Class 2 (2023 and 2024).

**Supporting Information 2** Figure S2: Longitudinal changes in student confidence in the knowledge of neuroscience content across course duration.

## Data Availability

The datasets that support the findings of this study are available in the supporting information of this article.
