# Supplementary material for: From Uncertainty to Competence: A Longitudinal Study of Confidence Development in Occupational Therapy Education
Source: Occup Ther Int. 2025 Dec 23;2025:1797008. doi: 10.1155/oti/1797008 (PMC12723319; doi:10.1155/oti/1797008)
Supplement: Supplementary file 1 — Supporting Information 1 Figure S1: Student self‐reported confidence in neuroscience‐related competencies across Class 1 and Class 2 (2023 and 2024). [file OTI-2025-1797008-s001.pdf]

Figure Title:
Supplemental Figure 1. Student Self-Reported Confidence in Neuroscience-Related Competencies Across Class 1 and Class 2 (2023 and 2024)

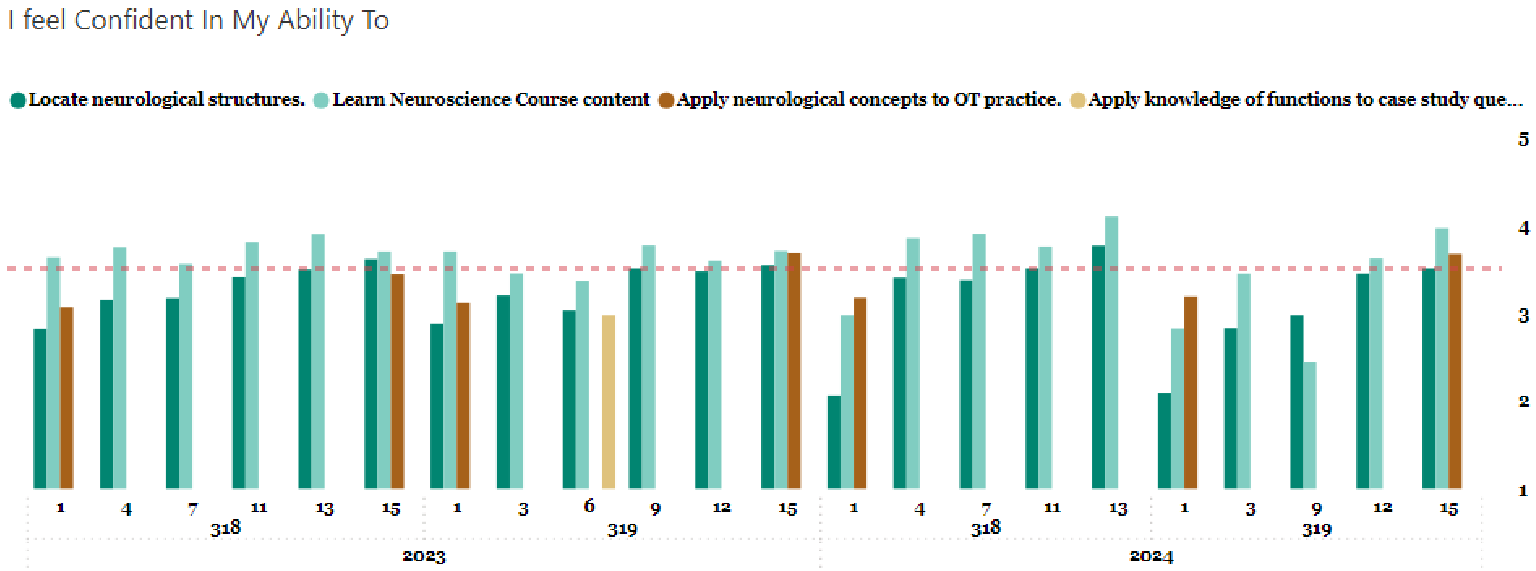

Figure Caption:
Average student confidence levels in four neuroscience-related competencies measured at weekly intervals throughout the course in Class 1 and Class 2 (2023 and 2024). Bars represent confidence in: locating neurological structures (dark teal), learning neuroscience course content (light teal), applying neurological concepts to occupational therapy practice (orange), and applying knowledge of functions to case study questions (lightest teal). Responses were rated on a 5-point Likert scale (1 = lowest confidence, 5 = highest confidence). The x-axis indicates the week of the course when surveys were administered. The dashed horizontal line indicates a confidence level of 3.5.
