# Supplementary material for: From Uncertainty to Competence: A Longitudinal Study of Confidence Development in Occupational Therapy Education
Source: Occup Ther Int. 2025 Dec 23;2025:1797008. doi: 10.1155/oti/1797008 (PMC12723319; doi:10.1155/oti/1797008)
Supplement: Supplementary file 2 — Supporting Information 2 Figure S2: Longitudinal changes in student confidence in the knowledge of neuroscience content across course duration. [file OTI-2025-1797008-s002.pdf]

Supplemental Figure 2. Longitudinal Changes in Student Confidence in Knowledge of Neuroscience Content Across Course Duration.

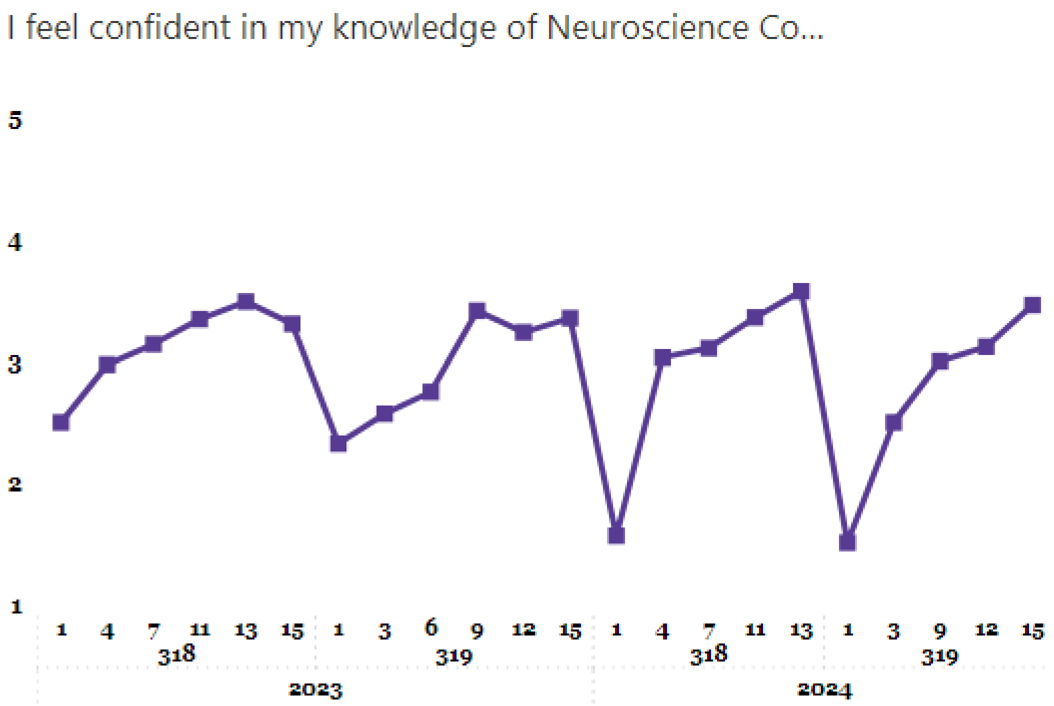

Figure Caption:
Student self-reported confidence in knowledge of neuroscience content measured weekly throughout the course in 2023 and 2024 cohorts. Confidence was rated on a 5-point Likert scale (1 = lowest confidence, 5 = highest confidence). The x-axis indicates the week of the course when surveys were administered. Notable trends include lower confidence levels at the beginning of the course (Week 1) with progressive improvement toward higher confidence by the final week (Week 15). Temporary dips in confidence observed mid-semester may correspond to exam periods or introduction of particularly challenging content.
